# Carotid Multicontrast ATherosclerosis CHaracterization (MATCH) in a single scan

**DOI:** 10.1186/1532-429X-16-S1-P46

**Published:** 2014-01-16

**Authors:** Zhaoyang Fan, Yibin Xie, Wei Yu, Qi Yang, Xiaoming Bi, Yutaka Natsuaki, Gerhard Laub, Jing An, Zhaoqi Zhang, Kuncheng Li, Zhanming Fan, Debiao Li

**Affiliations:** 1 Biomedical Sciences, Cedars-Sinai Medical Center, Los Angeles, California, USA; 2 Bioengineering, University of California, Los Angeles, California, USA; 3 Radiology, Anzhen Hospital, Capital Medical University, Beijing, China; 4 Radiology, Xuanwu Hospital, Capital Medical University, Beijing, China; 5 Siemens Healthcare R&D, Los Angeles, California, USA

## Background

The conventional MRI protocol for the characterization of atherosclerotic plaques involves a series of scans that provide multiple contrast weightings for resolving high-risk plaque characteristics [1]. However, mis-registration between image sets due to the inter-scan motion often compromises evaluation accuracy and image interpretation is expertise dependent [2]. The aim of this work was to develop a 3D MRI technique that acquires multiple image sets in a single scan with distinct contrast weightings.

## Methods

The developed MATCH sequence utilizes a low-flip-angle gradient echo-based MRI acquisition combined with magnetization preparative schemes, and multiple co-registered 3D image sets are collected in an interleaved scan with 4 TRs per cycle: the 1st TR provides hyper T1-weighted (T1w) contrast by using a nonselective inversion pulse and a blood-suppressing flow-sensitive dephasing (FSD) preparation [3]; the 2nd TR provides gray-blood lumen arising from both blood T1-recovery and in-flow fresh blood; the 3rd TR is for signal recovery without readout events, followed by the 4th TR for T2-weighted (T2w) contrast by using a long-duration FSD preparation. The three contrasts aims to identify the intra-plaque haemorrhage (IPH), juxtaluminal calcification (CA), loose matrix (LM), and potentially lipid core, respectively. The technique was optimized based on computer simulations and healthy volunteer studies and then evaluated on patients (n = 8) with carotid plaques on a 3T system (Siemens Verio). Imaging parameters include: 55-62 segments per TR of 1200 ms, flip angle = 8°, in-plane resolution = 0.55-0.63 mm, slice thickness = 2 mm, 18 slices, CHESS fat saturation, inversion time delay = 460-480 ms, m_1 _= 945 mTms^2^/m, FSD/T2 duration = 18/40 ms, centric reordering, iPAT = 2, scan time = 5-6 min. For comparison, spatially matched T1-w/T2-w TSE and TOF imaging were performed.

## Results

**Figure 1:**
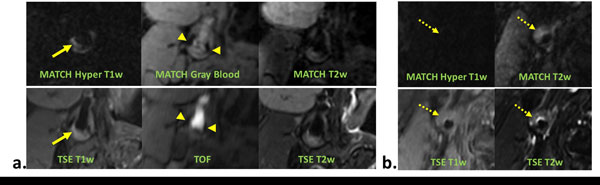
**Representative images obtained using the MATCH and conventional protocols in two patients (a. and b.)**. The major plaque components including intraplaque haemorrhage (arrows), juxtaluminal calcification (arrowheads), and loose matrix (dashed arrows) are better depicted on MATCH images.

**Figure 2:**
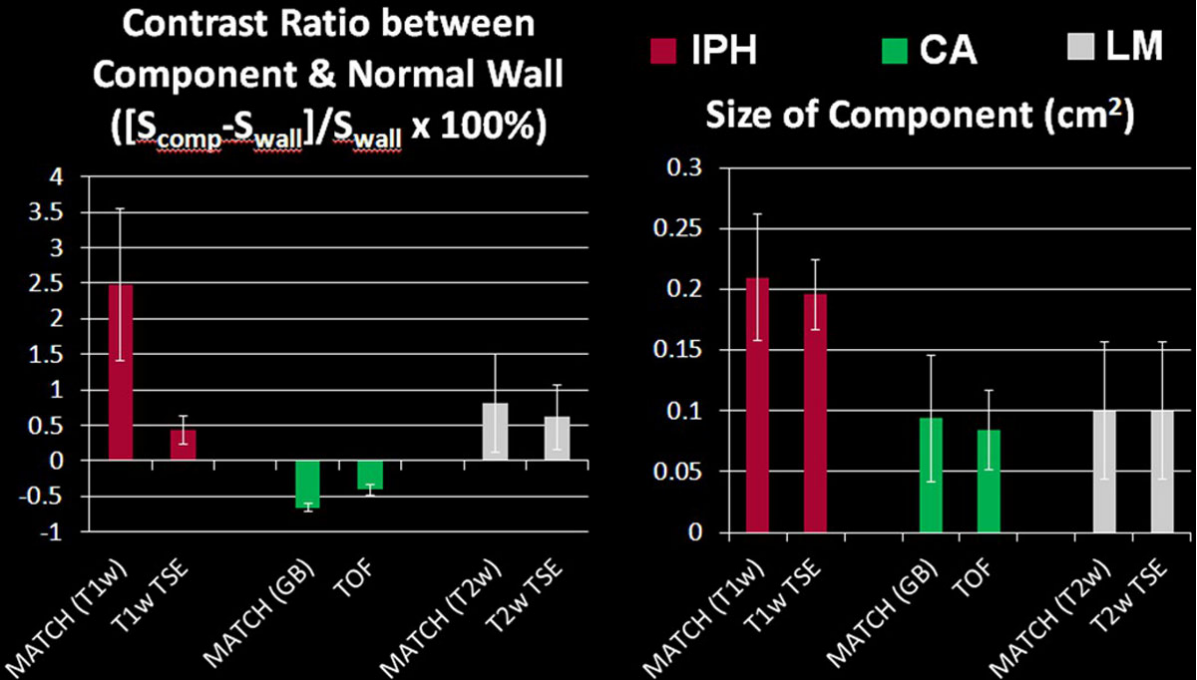
**Quantitative comparison between the MATCH and conventional protocols**. MATCH yielded better contrast ratio between each of the target components and the normal vessel wall. The size of each of the components measured using the two protocols were comparable.

A total of 12 locations with one of plaque components were assessed. With the MATCH acquisition, IPH (Figure 1a arrows) appeared hyper-intense on the hyper-T1w image set, CA (Figure 1a arrowheads) appeared as focal signal voids on gray-blood image set, and LM (Figure 1b dashed arrow) appeared hyper-intense on T2w but not on hyper-T1w. (Figure 1a &1b). Compared with the conventional protocol, MATCH yielded better contrast ratio between each of the target components and the normal vessel wall, markedly facilitating their identification (Figure 2). No appreciable difference in the size of components was observed between the two protocols.

## Conclusions

MATCH is a promising technique for an expedite and accurate characterization of carotid plaques. A large-scale patient validation is currently underway, using histology specimens as reference. Further technical improvements in spatial resolution and imaging speed will strengthen its clinical value.

## Funding

NIH HL096119, AHH 11POST7650043.

